# Optimization of Phlorizin Extraction from Annurca Apple Tree Leaves Using Response Surface Methodology

**DOI:** 10.3390/antiox11101933

**Published:** 2022-09-28

**Authors:** Maria Maisto, Vincenzo Piccolo, Ettore Novellino, Elisabetta Schiano, Fortuna Iannuzzo, Roberto Ciampaglia, Vincenzo Summa, Gian Carlo Tenore

**Affiliations:** 1 Department of Pharmacy, University of Naples Federico II, via Domenico Montesano 59, 80131 Naples, Italy; 2 Faculty of Medicine, University Cattolica, Largo Agostino Gemelli, 00168 Rome, Italy

**Keywords:** waste product, antioxidant activity, response surface methodology, antidiabetic activity, phlorizin

## Abstract

Phlorizin is a plant-derived molecule with relevant anti-diabetic activity, making this compound a potential functional component in nutraceutical formulations for the management of glycemia. It is noteworthy that promising sources for the extraction of phlorizin include apple tree leaves, a by-product of apple fruit production. The main aim of this study was to optimize the extraction process of phlorizin from Annurca apple tree leaves (AALs) using response surface methodology (RSM), and to determine the potential nutraceutical application of the obtained extract. The results of the RSM analysis indicate a maximum phlorizin yield of 126.89 ± 7.579 (mg/g DW) obtained under the following optimized conditions: MeOH/H_2_O, 80:20 + 1% HCOOH as the extraction solvent; 37.7 °C as the extraction temperature; and 170 min as the time of extraction. The HPLC-DAD-HESI-MS/MS analysis performed on the extract obtained under such conditions, named optimized Annurca apple leaves extract (OAALE), led to the identification of twenty-three phenolic molecules, with fifteen of them quantified. To explore the nutraceutical potential of OAALE, the in vitro antioxidant activity was evaluated by DPPH, ABTS, and FRAP assays, resulting in 21.17 ± 2.30, 38.85 ± 0.69, and 34.14 ± 3.8 μmol Trolox equivalent/g of extract, respectively. Moreover, the IC_50_ of 0.330 mg/mL obtained from the advanced glycation end-product inhibition assay, further supported the antidiabetic potential of OAALE.

## 1. Introduction

Phlorizin (phloretin-2-*O*-β-D-glucopyranoside) is the glucoside of phloretin, a member of dihydrochalcones, which are a family of bicyclic flavonoids. It was first isolated by a French scientist from apple tree bark in 1835 [1]. This molecule was largely studied for its multiple health effects, such as its anti-inflammatory, antioxidant, anticancer, and antibacterial activities [2]. Particularly, phlorizin plays an important role as a dietary polyphenol that is able to regulate glucose homeostasis by reducing intestinal glucose uptake [2]. More specifically, in diabetic rats, phlorizin was shown to inhibit intestinal and renal glucose uptake via sodium-dependent glucose transporters (SGLTs), resulting in a reduction in hyperglycemia without altering insulin secretion [3]. Other studies reported that phlorizin was not only able to reduce glucose plasma levels, but it also improved lipid metabolism [4], accelerated liver glycogen synthesis [5], decreased hepatic gluconeogenesis [6], and exerted hypoglycemic effects in type 2 diabetes mellitus mice [6].

The main natural sources of phlorizin are the plants of the Malus genus, although it also reaches a valuable concentration in other plant species, such as *Punica granatum* (pulp) [7], *Polygonum cuspidatum* (flower), *Prunus persica* (pulp) [8], *Rosa canina* (flesh) [9], *Vaccinium vitis-idaea* (flesh) [10], and *Vaccinium macrocarpon* [11]. Specifically, phlorizin is not equally distributed in all parts of the apple tree, however, it reaches its maximum concentration in the non-edible parts of the plant, e.g., leaves [12], twigs [13], root bark, seeds [14], and unripe fruits [15]. Phloretin, and its glucoside phlorizin, are found to be the major phenolic compounds in apple leaves, reaching a concentration ranging from 5.4% to 14% of leaf dry weight (DW) [12] Moreover, the phlorizin content of leaves seems to be less affected by some variables, such as apple cultivar or harvesting period, than its aglycone, making phlorizin concentration stable over time and in the type of apple cultivar analyzed.

Considering the conventional method of cultivation, during the pruning period in summer, unripe fruits and leaves are harvested to improve the quality of fruit production, resulting in a high amount of non-utilized apple leaves that would be classified as agro-food waste materials [12]. As widely reported, the interest of the nutraceutical industry in the reutilization of agro-food waste products is progressively increasing, since they represent still rich sources of biologically active compounds that can be conveniently used for the formulation of food supplements [16]. In this context, apple leaves may be considered an excellent source of bioactive compounds, especially phlorizin, their major phenolic component. Currently, the main apple-derived waste product reutilized by nutraceutical industries is root bark. Interestingly, compared to root barks, apple leaves have a similar dihydrochalcones content and are also produced in higher amounts and in every cycle of cultivation. Therefore, these byproducts could be considered a more convenient alternative raw material for the formulation of nutraceutical products rich in phlorizin. Due to the aforementioned biological activities ascribed to phlorizin, several extraction methods have been developed to optimize the extraction yield of this molecule from plant materials. In this regard, similarly to other polyphenolic compounds, the hydrochloric mixture is considered the more exhaustive solvent for its extraction [2].

Annurca apple is the only apple cultivar native to Southern Italy, listed as a Protected Geographical Indication (PGI) product by the European Council (Commission Regulation (EC) No.417/2006)). Annurca polyphenolic fraction is largely studied for its beneficial effects on the control and management of cholesterol plasma levels in healthy and mildly hypercholesterolemic subjects [17]. On the other hand, there has been a lack of studies regarding the chemical characterization of Annurca apple leaves (AALs) in the scientific literature. Moreover, compared to other apple cultivars, both local (native of the same region of Annurca apple, i.e., Rosa di Serino, Limoncella) and commercial ones (Pink Lady and Golden Delicious), Annurca apple showed the highest title in dihydrochalcones, and this trend would also be reproduced in the leaves [18,19].

In light of these considerations, the main goal of the present study was to investigate the potential of Annurca apple tree leaves (AALs) as a source of phenolic compounds, especially phlorizin. Moreover, the response surface methodology (RSM) was used to reach the maximum phlorizin extraction rate from AAL. After the determination of the optimum extraction condition (OEC), the polyphenolic composition of the extract obtained in OEC, named OAALE (Optimized Annurca Apple Leaves Extract), was investigated and its in vitro antioxidant and antidiabetic activities were studied.

## 2. Materials and Methods

### 2.1. Reagents

All chemicals, reagents, and standards used were analytical or LC–MS grade reagents. The water was treated in a Milli-Q water purification system (Millipore, Bedford, MA, USA) before use. Catechin (purity ≥ 98% HPLC), procyanidin B1 (purity ≥ 90% HPLC), procyanidin B2 (purity ≥ 90% HPLC), procyanidin B3 (purity ≥ 95% HPLC), procyanidin C1 (purity ≥ 90% HPLC), chlorogenic acid (purity ≥ 95% HPLC), caffeic acid (purity ≥ 98% HPLC), syringic acid (purity ≥ 98% HPLC), gallic acid (purity ≥ 98% HPLC) rutin (purity ≥ 94% HPLC), p-coumaric acid (purity ≥ 98% HPLC), epicatechin (purity ≥ 98% HPLC), ferulic acid (purity ≥ 99% HPLC), quercetin 3-*O*-glucoside (purity ≥ 98% HPLC), kaempferol 3-*O*-glucoside (purity ≥ 90% HPLC), quercetin (purity ≥ 98% HPLC), and the reagents for in vitro studies were purchased from Sigma-Aldrich (Milan, Italy).

### 2.2. Sample Collection and Extraction Protocol

AAL were harvested in October 2021 from the orchards of “Giaccio Frutta” society (Vitulazio, Caserta, Italy, 41°100 N–14°130 E). The AALs were frozen at −80 °C, lyophilized, and ground to obtain a homogeneous powder that constituted the production batch used for the experiments. As reported in Table 1, for the optimization of phlorizin extraction protocol from AALs, different extraction times (30, 60, 120, 240 min), solvent compositions (80% aqueous methanol solution containing 0.1, 1, or 5% formic acid), incubation temperatures (30, 35, and 45 °C), with and without a sonication stage of 30 min, were opportunely combined. According to the general extraction protocol applied, 250 mg of AALs were treated with 2 mL of extraction solvent, as previously optimized by Othman et al. [12] the mixture obtained was left in incubation at selected temperatures and times on an orbital shaker. At the end of the extraction time, where expected, 30 min of sonication (continuous operative mode, 150 W Power, 40 kHz Frequency; Branson Fisher Scientific 150E Sonic Dismembrator) was performed. After that, the samples were centrifuged for 5 min at 12,000× *g*. The supernatants were filtered with a 0.22 µm nylon filter (Cell Treat, Shirley, MA, USA) and stored at −20 °C until analysis. All extractions were performed in triplicate.

### 2.3. HPLC Analyses of Samples

#### 2.3.1. Qualitative Polyphenolic Composition by HPLC-DAD-HESI-MS/MS

An HPLC DIONEX UltiMate 3000 (Thermo Fisher Scientific, San Jose, CA, USA) equipment, coupled with an autosampler, a binary solvent pump, a diode-array detector (DAD), and an LTQ XL mass spectrometer (Thermo Fisher Scientific, San Jose, CA, USA), were used for the analysis. The chromatographic analysis was performed according to Maisto et al., with slight modifications [20]. The separation conditions were as follows: column temperature was set at 35 °C, the injection volume was 5 µL, and the flow rate was set at 1 mL/min. The selected column was the Kinetex^®^ C18 column (250 mm × 4.6 mm, 5 µm; Phenomenex, Torrance, CA, USA). The mobile phases were water at 0.1% formic acid (A) and acetonitrile at 0.1% formic acid (B). Elution was performed according to the following conditions: 0–3 min hold at 5% solvent B, from 5% (B) to 40% (B) in 20 min and 95% (B) in 10 min, followed by 5 min of maintenance; for the remaining 10 min, the column was equilibrated to the initial conditions. Regarding the mass parameters, the source was a heated electrospray interface (HESI), operated in negative ionization with full scanning (FS) and data-dependent acquisition (DDA). Phenolic acids, hydroxycinnamic acids, flavanols, and flavanones were monitored at 280 nm, while flavonols were monitored at 360 nm. Collision-induced fragmentation was made using argon, with a collision energy of 35.0 eV. The ion source was set using the following parameters: sheath gas flow rate: 30; auxiliary gas flow rate: 10; capillary temperature: 320 °C; source heated temperature: 150 °C; source voltage: 3.5 kV; source current: 100 µA; capillary voltage: 31 V; and tube lens: 90 V.

#### 2.3.2. Quantitative Polyphenols Analysis by HPLC-DAD-FLD

The quantitative analysis of OECE was performed with the HPLC Jasco Extrema LC-4000 system (Jasco Inc., Easton, MD, USA), equipped with an autosampler, a binary solvent pump, a diode-array detector (DAD), and a fluorescence detector (FLD). The chromatographic analysis was performed according to our previously developed method [20]. Procyanidins were detected by a fluorescence detector that was set with an excitation wavelength of 272 nm and an emission wavelength of 312 nm, while the phenolic acids, hydroxycinnamic acids, flavanols, and flavanones were acquired at 280 nm, and flavonols at 360 nm (Supplementary Materials Figures S1–S4). The analyses were performed at a flow rate of 1 mL/min, with solvent A (2% acetic acid) and solvent B (0.5% acetic acid in acetonitrile and water 50:50, *v*/*v*) using Kinetex^®^ C18 column (250 mm × 4.6 mm, 5 µm; Phenomenex, Torrance, CA, USA): 0–5 min of 10% (B), from 10% (B) to 55% (B) in 50 min and 95% (B) in 10 min, followed by 5 min of maintenance. Peak identifications were based on a comparison of retention times with analytical standards and standard addition to the samples. The quantitative analyses were performed using the calibration curve calculated with six different concentrations in a concentration range of 0.1–1000 ppm and triplicate injections at each concentration level.

### 2.4. Total Phenolic Content Determination

The total phenol content (TPC) was performed by Folin–Ciocalteau’s assay, using gallic acid as the reference standard (Sigma-Aldrich, St. Louis, MO, USA). Briefly, 0.1 mL of samples (appropriately diluted with water to achieve a measured absorbance value included the linear range of the spectrophotometer) were added in sequence: 0.5 mL of Folin–Ciocalteau’s (Sigma-Aldrich, St. Louis, MO, USA) reagent and 0.2 L of an aqueous solution of Na_2_CO_3_ 7% (*w*/*v*%), bringing the final volume to 10 mL with water. Then, the samples were mixed and left in incubation in the dark for 90 min. After the reaction time, the absorbance was acquired at 760 nm (Jasco Inc., Easton, MD, USA). All the samples were analyzed in triplicate and the concentration of total polyphenols was calculated in gallic acid equivalents (GAEs).

### 2.5. Antioxidant Activity

#### 2.5.1. DPPH^•^ Radical Scavenging Assay

The radical scavenging ability of the antioxidants in the sample was evaluated using the stable radical 2,2-diphenyl-1-picrylhydrazyl (DPPH) with a maximum absorbance at 517 nm. The analysis was performed by mixing 100 µL of each sample (opportunely diluted in extraction mixture) with 1000 µL of a methanol solution of DPPH (153 mmol/L). The mixture obtained was left in incubation in the darkness for 9 min of reaction time. The decrease in absorbance was evaluated using a UV–visible spectrophotometer (Beckman, Los Angeles, CA, USA). All determinations were performed in triplicate. DPPH^•^ inhibition was calculated according to the formula: [(A_i_ − A_f_)/A_c_] × 100, where A_i_ is the absorbance of the sample at t = 0, A_f_ is the absorbance of the sample after the reaction time and A_c_ was the absorbance of the control (1000 µL of a methanol solution of DPPH + 100 µL of methanol). The obtained results are expressed in µmol of Trolox (6-hydroxy-2,5,7,8-tetramethylchroman-2-carboxylic acid) equivalent (TE). Moreover, the results were also reported as EC_50_, which is the amount of antioxidant compound necessary to inhibit the initial DPPH^•^ concentration by 50% [21].

#### 2.5.2. Ferric Reducing/Antioxidant Power (FRAP) Assay

When a Fe^3+^-TPTZ complex is reduced to the Fe^2+^ ion by an antioxidant under acidic conditions, a blue color develops, with maximum absorbance at 593 nm [18]. Thereby, the antioxidant effect (reducing ability) of the sample was evaluated by monitoring the formation of a Fe^2+^–TPTZ complex with a spectrophotometer (Jasco Inc., Easton, MD, USA). The test was performed as reported by Benzie and Strain (1996) [22], with slight modifications. The Frap working solution was prepared by mixing 10 vol of 0.3 M acetate buffer, pH 3.6 (3.1 g sodium acetate and 16 mL glacial acetic acid), 1 vol of 10 mM TPTZ prepared in 40 mM HCl, and 1 vol of 20mM FeCl_3_. All the components of the working solutions were freshly prepared and used on the same day of preparation. Before performing the assay, all the solutions were brought to 37 °C. The amount of 2.85 mL of working solution was mixed with 0.15 mL diluted samples and incubated at 37 °C for 4 min. After the incubation time, the absorbance was acquired at 593 nm (Jasco Inc., Easton, MD, USA). The blank was represented by the only working solution. For the calculation of antioxidant activity, the blank absorbance value was subtracted from the absorbances of the samples. All analyses were performed in triplicate. A standard curve was plotted with Trolox, and the results are expressed as µmol TE.

#### 2.5.3. ABTS^•^ Radical Scavenging Assay

The assay relied on the capability of antioxidant molecules to react ABTS^•+^ radical (2,20-azinobis(3-ethylbenzotiazoline-6-sulfonate)), a chromophore with specific absorption at 734 nm. The test was performed according to the experimental protocol previously performed by Babbar et al. (2011) [23] with some modifications.

ABTS solution was prepared by mixing 2.5 mL of ABTS 7.0 mM ethanol solution and 44 µL of potassium persulfate 140 mM solution, which was left to incubate for at least 7 h, at 5 °C in darkness. After this time, to prepare the working solution, the obtained mixture was diluted with the ethanol–water solution until an absorbance value of 0.700 ± 0.05 was acquired at 754 nm (Jasco Inc., Easton, MD, USA). The assay was performed by mixing 1000 µL ABTS working solution with 100 µL of the sample opportunely diluted in the extraction solvent. The mixture was incubated for 2.5 min in the dark. After this time, the sample absorbances were read at 734 nm, with a visible discoloration of the sample with high antiradical activity. The control was prepared by replacing the sample with the same volume of ethanol. The radical inhibition was calculated according to the formula: [(A_i_ − A_f_)/A_c_] × 100, (2), where A_i_ is the absorbance of the sample at t = 0, A_f_ is the absorbance after 2.5 min, and A_c_ is the absorbance of the control at time zero. Trolox was used as a standard antioxidant. The results are expressed both as µmol of TE and EC_50_, which is the amount of antioxidant necessary to decrease the initial ABTS^•+^ concentration by 50% [21].

### 2.6. Advanced Glycation End-Product (AGE) Inhibition Assay

The inhibition of AGE generation by OAALE extract and the standard phenolic rutin was performed according to the method reported by Schiano et al. [15] with slight modifications. The amount of 500 µL of serial dilutions for each sample (0.075–70 mg/mL of final concentrations for OAALE and 0.05–2 mg/mL for rutin) prepared in distilled water were added to a working solution composed of 500 µL of bovine serum albumin (50 mg/L), 250 µL fructose (1.25 mol/L) and 250 µL of glucose (25 mol/L). All the elements of this reaction mixture were dissolved in phosphate buffer (200 mmol/L; pH 7.4) containing sodium azide (0.02% *w*/*v*). The mixture was incubated at 37 °C for 7 days. After this incubation time, the fluorescence was acquired at an excitation wavelength of 355 nm and an emission of 460 nm (Perkin-Elmer LS 55, Waltham, MA, USA). Distilled water was used as a negative control, while the blank was carried out by replacing the fructose and glucose with phosphate buffer. The inhibitory activity was expressed as a percentage of glycation inhibition (GI), using the following formula: GI (%) = [(F_s_ − F_sb_)/(F_c_ − F_cb_)] × 100, (4) where F_s_ is the fluorescence intensity in the presence of the sample; F_sb_ is the fluorescence intensity in the absence of fructose and glucose; F_c_ is the fluorescence intensity in the absence of sample; and F_cb_ is the fluorescence intensity in the absence of sample, fructose, and glucose. Finally, the results are reported as EC_50_.

### 2.7. Statistics

Unless otherwise stated, all experimental results are expressed as the mean ± standard deviation (SD) of three repetitions. Graphics and IC_50_ values determination were calculated using GraphPad Prism 8 software. The RSM optimization was performed with Minitab software version 21.1.0.

## 3. Results and Discussion

### 3.1. Optimisation of Phloridzin Extraction using RSM Model

**Figure 1:**
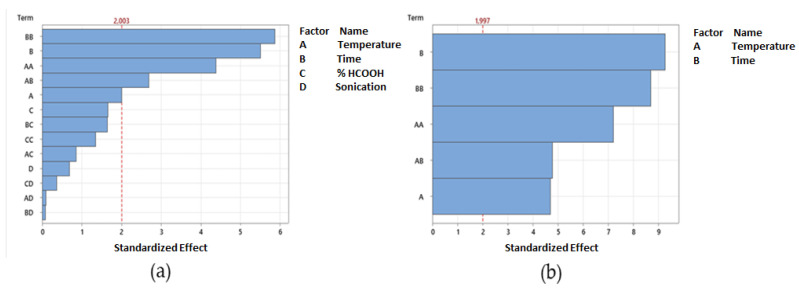
(**a**) Pareto chart of the total parameters analyzed (significative and not significative); and (**b**) Pareto chart of significative parameters only, i.e., temperature (A) and extraction time (B).

The choice to optimize the phlorizin extraction conditions in MeOH 80% was due to the capacity of this solvent to reach the maximum extraction rate not only of phlorizin, but also of total polyphenols [2,20,23]. The temperature was kept constantly below 40 °C to avoid the temperature-dependent decomposition of polyphenols during the extraction process. Generally, the stability of polyphenols at high-temperature values depends on the class of polyphenols considered and, obviously, on their chemical structure. Specifically, it was well accepted that the polyphenols concentration was significantly stable (*p* < 0.05) during the extraction process at a temperature lower than 40 °C [24]. The experimental data show that the phlorizin concentration ranged from 70.80 mg/g (*p* < 0.001) (60 min, 25 °C, 1% HCOOH with 30 min of sonication) to 141.59 mg/g (120 min, at 35 °C, +1% HCOOH, without sonication). Initially, four independent commonly modified factors, i.e., extraction time (30, 60, 120, and 240 min), temperature (45, 35, and 25 °C), and % acid in the extraction solvent (5, 1, and 0.1% of formic acid), combined with or without a single cycle of sonication (30 min), were selected for the optimization of phlorizin yield in the hydroalcoholic solvent. Considering the independent factors analyzed, according to preliminary ANOVA analysis, only the extraction temperature (A) and extraction time (B), without sonication assistance, were significantly correlated with the phlorizin extraction rate, as explained in the Pareto-chart graphic with α = 0.05 (Figure 1).

Based on the statistical results of model fitting, the best model to optimize the phlorizin output would be by reducing the statistical analysis to two-factor interaction (2FI, i.e., A and B) (Figure 1). The multiple regression analysis of phlorizin values showed that the model was significant (*p* < 0.0001), did not present a lack of fit *(p* = 0.182), and a percentage predictivity of the model was of 73.56% (R-sq 77.58%, R-sq(adj) 75.88%; R-sq(pre) 73.41%). Second-order quadratic polynomial models were found to be adequate to describe the effect of the two independent and significative factors on the phlorizin output, as described by Equation (1), in terms of uncoded units.

**Figure 2:**
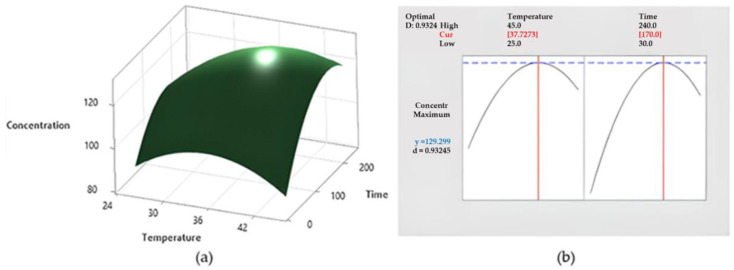
(**a**) Surface plot of phlorizin concentration *cv* time and temperature; and (**b**) multiple response prediction analysis.

Phlorizin Concentration = −57.9 + 8.46 A + 0.3209 B − 0.1254 A*A − 0.001609 B*B + 0.00597 A*A(1)

where factors A and B are the temperature and extraction time, respectively. According to the process model (Equation (1)), factors A and B affected the phlorizin yield in different ways. Specifically, temperature (A) was reported in the polynomial Equation (5) times vs. three times of the extraction time (B), highlighting that the extraction temperature played a predominant role in influencing the phlorizin yield. Moreover, as described by Equation (1), the increase in factor B may lead to a decrease in phlorizin yield. It was well accepted that the extended extraction time can damage the extracted phlorizin and degrade extract quality [25]. The dominant role of temperature in influencing the polyphenols extraction rate was largely described [25]. A high extraction temperature indeed decreases the viscosity of the extraction medium, which helps the solvent penetrate the plant matrix, resulting in faster kinetics [26]. Moreover, the increment in solvent temperature may decrease the surface tension and, consequently, enhances the wetting of the plant particles, leading to a higher extraction yield [27] contrastingly, as confirmed by Equation (1), the temperature value must be kept within some limits, beyond which it determines the degradation of polyphenols. The same effect was also shown by the 3D response surface (Figure 2a). The predictive model studied indicates that the theoretical condition to achieve the maximum phlorizin extraction consisted of the hydroalcoholic extraction (MeOH/H_2_O, 80:20 + 1% HCOOH), conducted at 37.7 °C for 170 min, as reported in Figure 2. These variables were combined to set up another new extraction from LAA to verify and confirm the theoretical phlorizin concentration (129.29 mg/g) described by the multiple response prediction analysis.

Therefore, the experimental phlorizin concentration obtained in the extract, by the application of these optimized conditions, was 126.89 ± 7.579, with an EA of 101.89%. Because of the low absolute error values achieved by the comparison between observed and predicted values, the proposed model may be used to predict the experimental value.

### 3.2. Quantitative Polyphenols Analysis by HPLC-DAD-FLD

Chromatographic analysis for the quantification of OAALE polyphenolic composition was performed as previously described in Section 2.3. The HPLC-DAD-FLD analysis resulted in the identification and quantification of 15 different selected phenolic compounds, counting flavanols, procyanidins, phenolic acids, and flavonols. The obtained results are reported in Table 2. As expected, phlorizin and phloretin were some of the most abundant and representative polyphenols contained in OAALE. Beyond dihydrochalcones, the second most representative class of polyphenols In OAALE were flavanols. Quercetin-3-*O*-glucoside and Kaempferol-3-*O*-glucoside reached a valuable concentration in OAALE of 3.27 and 20.09 mg/g, respectively. Similarly, Othman et al. reported a relevant flavanol content in the apple leaf extract. Moreover, among the flavanols detected by the same researchers, quercetin-3-*O*-rhamnoside was the most abundant polyphenolic compound in extracts obtained from the leaves of different apple cultivars. Chlorogenic acid was the major phenolic acid detected in OCE, followed by caffeic acid, 0.209 and 0.0785 mg/g of dry weight, respectively. Additionally, other studies related to apple leaf extracts also reported chlorogenic acid as the most abundant phenolic acid [28]. As regards the dimeric procyanidin content, a higher amount was observed for procyanidin B2 (0.454 mg/g), followed by procyanidin B1 and B3. Our results are in line with other evidence about the procyanidin B2 as the most abundant procyanidin compound in apple leaf extract [29].

### 3.3. Qualitative Polyphenols Analysis by HPLC-HESI-MS/MS

OCE polyphenolic composition was characterized by HPLC-HESI-MS/MS, as reported in Section 2.3. Based on a comparison with the literature data, 23 compounds were putatively identified (Table 3). Compound **1** showed a [M-H]^−^ ion at *m*/*z* 197. The base peak ion at *m*/*z* 182 [M-H-CH_3_]^−^ and the fragment ions of its tandem mass spectrum at *m*/*z* 179 [M-H-H_2_O]^−^, *m*/*z* 153 [M-H-CO_2_]^−^ and *m*/*z* 138 [M-H-CO_2_-CH_3_]^−^, suggested the presence of a carboxylic acid, a methoxy and a phenolic group. According to the mass fragmentation pattern, compound **1** was identified as syringic acid [30]. Compounds **2** and **4** displayed a [M-H]^−^ ion at *m*/*z* 163 and a base peak ion at *m*/*z* 119 [M-H-CO_2_]^−^. The fragment ions at *m*/*z* 145 [M-H-H_2_O]^−^ and at *m*/*z* 135 [M-H-CO]^−^ indicated the presence of the hydroxycinnamic acid scaffold and a phenol group. In agreement with the literature data, compounds **2** and **4** were annotated as *p*-coumaric acid isomers [31]. Compound **3** showed a [M-H]^−^ ion at *m*/*z* 353 and was putatively identified as a caffeoylquinic acid. The base peak ion at *m*/*z* 191 [M-H-CA]^−^ and the fragment ion at *m*/*z* 179 [M-H-QA]^−^ were due to the loss of the caffeic acid and the quinic acid group, respectively. By comparison with an authentic analytical standard, compound **3** was identified as chlorogenic acid [32]. Caffeic acid (**5**) displayed a [M-H]^−^ ion at *m*/*z* 179. The base peak ion at *m*/*z* 135 [M-H-CO_2_]^−^ and the fragment ions at *m*/*z* 161 [M-H-H_2_O]^−^ and *m*/*z* 107 [M-H-CO-CO_2_]^−^ highlighted the linkage of a carboxylic acid and a phenolic acid. One procyanidin dimer B-type linkage (**6**) showed a [M-H]^−^ ion at *m*/*z* 577 and a base peak ion at *m*/*z* 425 [M-H-C_8_H_8_O_3_]^−^, due to the RDA fission. The fragment ions at *m*/*z* 451 [M-H-C_6_H_6_O_3_]^−^, at *m*/*z* 289 [M-H-C_15_H_12_O_6_]^−^ and at *m*/*z* 287 [M-H-C_15_H_14_O_6_]^−^ were produced by the HRF and the QM cleavage, respectively. By comparison with the authentic analytical standard, compound **6** was identified as procyanidin B2 [31]. Compound **7** displayed a [M-H]^−^ ion at *m*/*z* 289 and was putatively identified as epicatechin. The base peak ion at *m*/*z* 245 [M-H-C_2_H_4_O]^−^ and the fragment ion at *m*/*z* 137 [M-H-C_8_H_8_O_3_]^−^, due to the RDA fragmentation, were in agreement with the literature data [33]. Two 4-*O*-coumaroylquinic acid isomers (compounds **8** and **9**) were tentatively identified. They showed a [M-H]^−^ ion at *m*/*z* 337 and three fragment ions at *m*/*z* 191 [QA-H]^−^, at *m*/*z* 173 [QA-H-H_2_O]^−^ and at *m*/*z* 163 [M-H-QA]^−^, due to the fragmentation of the quinic acid moiety. However, the base peak ion at *m*/*z* 173 [QA-H-H_2_O]^−^ indicated the linkage between quinic acid and coumaric acid moieties with the 4-OH group. Therefore, compounds **8** and **9** were putatively identified as 4-*O*-coumaroylquinic acid isomers [34]. Four quercetin *O*-hexoside isomers (compounds **10**, **14**, **17,** and **20**) were putatively detected. They showed an [M-H]^−^ ion at *m*/*z* 463 and a base peak ion at *m*/*z* 301 [M-H-Hex]^−^ due to the fragmentation of the hexoside group. The fragments at *m*/*z* 445 [M-H-H_2_O]^−^ and at *m*/*z* 179 [M-H-Hex-C_7_H_6_O_2_]^−^, due to the RDA fragmentation, confirmed the presence of the flavonol scaffold and agreed with literature data [35]. Two quercetin *O*-rutinoside isomers (compounds **11** and **13**) were tentatively identified and displayed a [M-H]^−^ ion at *m*/*z* 609. The base peak ion at *m*/*z* 301 [M-H-Glu-Rha]^−^, due to the loss of the disaccharide group, and the fragment ions at *m*/*z* 463 [M-H-Rha]^−^ and at *m*/*z* 179 [M-H-Glu-Rha-C_7_H_6_O_2_]^−^, which derived from the RDA fragmentation, were consistent with the literature data [35]. However, compound **13** was identified as rutin by comparison with the authentic analytical standard. Compound **12** displayed a [M-H]^−^ ion at *m*/*z* 433. The base peak ion at *m*/*z* 271 [M-H-Hex]^−^ and the fragment ions at *m*/*z* 313 [M-H-C_4_H_8_O_4_]^−^ and at *m*/*z* 151 [M-H-Hex-C_8_H_8_O]^−^, due to the RDA fragmentation, allowed the identification of the flavanone scaffold. Therefore, compound **12** was annotated as naringenin *O*-hexoside [35]. Compound **15** showed a [M-H]^−^ ion at 431. The base peak ion at *m*/*z* 269 [M-H-Hex]^−^ and the fragment ion at *m*/*z* 311 [M-H-C_4_H_8_O_4_]^−^ are derived from the cleavage of the hexoside group and the RDA fragmentation, respectively. Based on the tandem mass spectrum and by comparison with an analytical standard, compound **15** was identified as apigenin 7-*O*-glucoside. Compound **16** displayed a [M-H]^−^ ion at *m*/*z* 593 and was annotated as kaempferol *O*-rutinoside. The base peak ion at *m*/*z* 285 [M-H-Pent-Hex]^−^ and the fragment ions at *m*/*z* 327 [M-H-Pent-C_4_H_8_O_4_]^−^ and *m*/*z* 257 [M-H-Pent-Hex-CO]^−^ confirmed the linkage of the disaccharide rutinose and the aglycone kaempferol [36]. Compound **18** showed a [M-H]^−^ ion at *m*/*z* 433. The base peak ion at *m*/*z* 301 [M-H-Pent]^−^ and the fragment ion at *m*/*z* 179 [M-H-Pent-C_7_H_6_O_2_]^−^ allowed us to identify the presence of the pentoside group and the flavanol scaffold. Based on the tandem mass spectrum, compound **18** was annotated as quercetin *O*-pentoside. Quercetin O-rhamnoside (**19**) displayed an [M-H]^−^ ion at *m*/*z* 447 and a base peak ion at *m*/*z* 301 [M-H-Rha]^−^ for the loss of the rhamnoside unit. The fragment ions at *m*/*z* 429 [M-H-H_2_O]^−^, *m*/*z* 179 [M-H-Rha-C_7_H_6_O_2_]^−^ and *m*/*z* 151 [M-H-Rha-C_8_H_6_O_3_]^−^ confirmed the presence of the flavanol scaffold and are consistent with the literature data. Compound **21** displayed a [M-H]^−^ ion at *m*/*z* 435. The base peak ion at *m*/*z* 273 [M-H-Hex]^−^ and the prominent fragment ion at *m*/*z* 167 [M-H-Hex-C_7_H_6_O]^−^ indicated the presence of the chalcone scaffold and the linkage of the hexoside group. Based on these data and by comparison with an analytical standard, compound **21** was identified as phloridzin. Kaempferol 3-*O*-rhamnoside (**22**) showed a [M-H]^−^ ion at *m*/*z* 431. Its tandem mass spectrum displayed a base peak ion at *m*/*z* 285 [M-H-Rha]^−^ and two fragment ions at *m*/*z* 327 [M-H-C_4_H_8_O_3_]^−^ and *m*/*z* 179 [M-H-Rha-C_7_H_6_O]^−^, due to the loss of the sugar moiety and RDA fragmentation [36]. The identity of compound **22** was confirmed by comparison with the analytical standard. Compound **23** displayed a [M-H]^−^ ion at *m*/*z* 273. Its tandem mass spectrum is characterized by a base peak ion at *m*/*z* 167 [M-H-C_7_H_6_O]^−^ and a fragment ion at *m*/*z* 125 [M-H-C_9_H_8_O_2_]^−^, which is linked to the presence of the chalcone moiety. Based on these data and by comparison with an analytical standard, compound **23** was identified as phloretin [36].

### 3.4. Total Polyphenols and In Vitro Antiradical Activity of OAALE

The antiradical potential of apple leaves, as vegetal matrices [39], considering their well-accepted relation with diabetes and oxidative stress [40], prompted us to evaluate the total phenolic content (TPC) and the in vitro antiradical activity of OAALE. Thus, to obtain a general overview of its total polyphenolic content, Folin–Ciocalteau’s test was performed on OAALE, resulting in 23.70 ± 1.23 mg GAE/g of Annurca apple leaves (AALs). As expected, the TPC of AALs was higher than the TPC of Annurca apple fruit (AAF), which was 1.94 mg/g of DW of whole fruit (peel and pulp) [41]. The calculation of antiradical activity was measured by the application of DPPH, ABTS, and FRAP assays on OAALE, as described in Section 2. Results are reported in Table 4.

**Figure 3:**
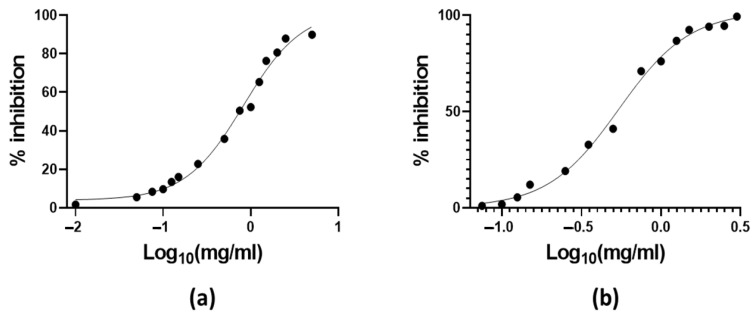
Antiradical activity of OAALE expressed as (**a**) EC_50_ of DPPH assay and (**b**) EC_50_ of ABTS assay. Values represent the mean ± standard deviation of triplicate reading.

Regarding the antiradical activity, OAALE has shown a higher relevant activity compared to AAF (antiradical activity, respectively, of 0.048 for ABTS, 0.01559 for DPPH, and 0.0266 µmol TE/g DW for FRAP) [41]. Moreover, in order to standardize the results of the various activities studied, the results of DPPH and ABTS assays were also calculated as EC_50_, which is the quantity of antioxidants necessary to decrease the concentration of the initial solution by 50% [21]. Figure 3 reported that the OAALE extract exhibited an EC_50_ of 0.828 mg/mL for the DPPH assay and 0.542 mg/mL for the ABTS assay. Therefore, these results would support the relevant potential application of OAALE as a source of antiradical agents, with the indubitable benefit of re-evaluating food waste. It is noteworthy that increasing evidence from in vitro and clinical trials indicates that oxidative stress may play a relevant role in the pathogenesis of diabetes. High levels of free radicals, and the concomitant decrease in antioxidant defense mechanisms, may lead to the injury of biological structures, which is recognized as the main pathological origin for the generation and development of diabetes-related complications [40].

### 3.5. In Vitro Antidiabetic Activity

**Figure 4:**
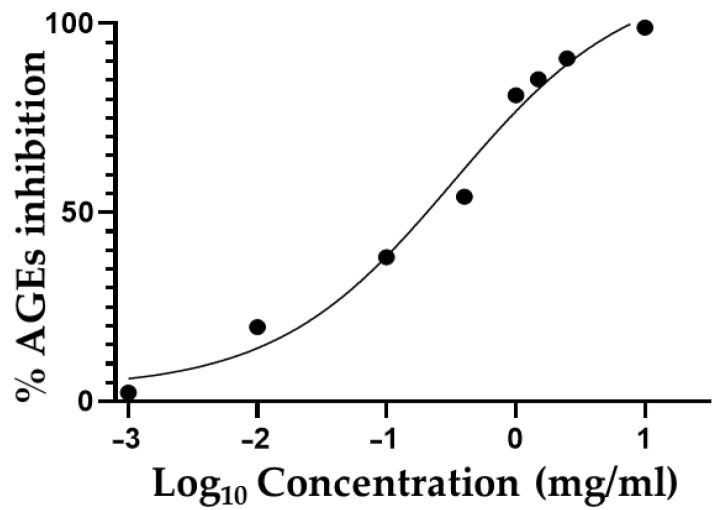
Inhibition of advanced glycation end-product formation (%) by OAALE. Values represent the mean ± standard deviation of triplicate readings.

Increasing evidence has identified the formation of advanced glycation end-products (AGEs) as a major pathogenic risk agent related to hyperglycemia and diabetes-related complications. It is also well known that the continuous AGEs accumulation in tissues and organs is directly linked to the development of chronic diabetic-related complications, such as retinopathy, nephropathy, neuropathy, and macrovascular disease [15,42]. AGEs are proteins or fats combined with blood sugars after exposure to a glycation process through the Maillard reaction [43]. These compounds are extremely and negatively stable and resistant to enzymatic activities, resulting in their relevant accumulation in different tissues, which may cause a remarkable morphological change in cell tissue, with a continuous deterioration of tissue structure and the alteration of their physiological function [15,42]. Therefore, the concentration-dependent inhibition of AGEs formation after the treatment with OAALE was evaluated, with the results reported in Figure 4. The calculated IC_50_ value was 0.330 mg/mL. In this regard, phlorizin and phloretin may be considered the main actors of OAALE potential valuable biological activity. In support of this hypothesis, these two molecules represent the main polyphenolic components of OAALE and, as reported in other studies, both of them demonstrated the inhibition of AGEs formation in a concentration-dependent manner, at a concentration range of 0.01–1.0 mM [44].

In this context, although dihydrochalcones were the most abundant molecules in OAALE, we also showed a valuable concentration of other classes of polyphenols (as reported in Section 3.2 and Section 3.3), which may contribute to the inhibition of AGEs formation. Specifically, polyphenol antiglycation properties are due to their capacity to stop the formation of a principal precursor of the Maillard reaction, the methylglyoxal (MGO) [45]. While phenolic acids and flavanols (e.g., gallic acid, p-coumaric acid, and epicatechin) described a direct inhibition mechanism by a reduction in the carbonyl groups of MGO, an indirect reaction with an MGO dicarbonyl moiety was reported [45,46]. Based on such a consideration, the IC_50_ of 0.330 mg/mL would be attributed to the synergic action of dihydrochalcones and other polyphenols contained in OAALE.

## 4. Conclusions

The previously described results indicate that AAL could be considered an excellent by-product source of bioactive compounds, especially phlorizin. Notably, the optimization of the extraction protocol conducted using the RSM methodology allowed us to evaluate the maximum extractable phlorizin amount contained in AAL (126.89 mg/g). The extract obtained under optimized conditions (OAALE) was also chemically characterized and its in vitro potential biological activity was tested. The promising results about the antioxidant activity and the inhibition of AGEs formation may suggest that AALs are a powerful functional ingredient, useful for the formulation of nutraceutical products for the management of diabetes disease. Further investigations about the beneficial potential exerted by the formulation in a diabetes model are required to assess the effective application in the management of this pathological condition. In addition, future perspectives include the possibility of performing a toxicological analysis aiming to exclude the possible residues of the means used to treat apple trees.

## Figures and Tables

**Table 1:** Independent variables and their values used for the model set.

Independent Variable	Factor Levels			
Incubation time (min)	30	60	120	240
% Acid in the extraction solvent	0.1	1	5	
Temperature (°C)	45	35	25	
Sonication	Yes	No		
Total runs	69			

**Table 2:** Quantitative analysis of OAALE determined by HPLC-DAD-FLD analysis.

Compound	Mean Value ± SD (mg/g)
Chlorogenic acid	0.2090 ± 0.0040
Caffeic acid	0.0785 ± 0.0013
*p*-Cumaric acid	0.0081 ± 0.0001
Procyanidin B1+B3	0.1634 ± 0.0003
Procyanidin B2	0.4540 ± 0.0080
Epicatechin	0.2000 ± 0.0037
Rutin	0.3510 ± 0.0010
Quercetin-3-*O*-glucoside	3.2740 ± 0.0010
Kaempferol-3-*O*-rhamnoside	0.1680 ± 0.0070
Kaempferol-3-*O*-glucoside	20.0970 ± 0.3820
Apigenin-7-*O*-glucoside	0.0081 ± 0.0001
Phloridzin	126.8900 ± 7.5790
Quercetin	0.0152 ± 0.0001
Phloretin	0.8650 ± 0.0070

Values are expressed in mg/g ± standard deviation (SD) of three repetitions. Procyanidins B1 and B3 peaks were partially overlapped and were quantified as a mixture of two compounds using the procyanidin B1 calibration curve.

**Table 3:** Polyphenolic composition of OAALE extracts determined by HPLC-HESI–MS/MS analysis.

No.	Compound	R_t_ (min)	UV–Vis (nm)	*m*/*z*	Diagnostic Fragment	Ref.
1	Syringic acid	9.56	210, 260	197	**182** [M-H-CH_3_]^−^, 179 [M-H-H_2_O]^−^, 153 [M-H-CO_2_]^−^, 138 [M-H-CO_2_-CH_3_]^−^	[30]
2	Coumaric acid isomer 1	11.17	215, 310	163	145 [M-H-H_2_O]^−^, 135 [M-H-CO]^−^, **119** [M-H-CO_2_]^−^	[31]
3	Chlorogenic acid	11.52	215, 295, 325	353	**191** [M-H-CA]^−^, 179 [M-H-QA]^−^, 173 [M-H-CA-H_2_O]^−^, 161 [M-H-QA-H_2_O]^−^	[32]
4	Coumaric acid isomer 2	11.71	210, 305	163	145 [M-H-H_2_O]^−^, 135 [M-H-CO]^−^, **119** [M-H-CO_2_]^−^, 101 [M-H-CO_2_-H_2_O]^−^	[31]
5	Caffeic acid	11.95	205, 280	179	161 [M-H-H_2_O]^−^, 151 [M-H-CO]^−^, **135** [M-H-CO_2_]^−^, 107 [M-H-CO-CO_2_]^−^	[32]
6	Procyanidin B2	12.35	210, 295	577	451 [M-H-C_6_H_6_O_3_]^−^, **425** [M-H-C_8_H_8_O_3_]^−^, 289 [M-H-C_15_H_12_O_6_]^−^, 287 [M-H-C_15_H_14_O_6_]^−^	[31]
7	4-*O*-Coumaroylquinic acid isomer 1	12.90	215, 310	337	319 [M-H-H_2_O]^−^, 191 [QA-H]^−^, **173** [QA-H-H_2_O]^−^, 163 [M-H-QA]^−^	[33]
8	4-*O*-Coumaroylquinic acid isomer 2	13.15	215, 310	337	319 [M-H-H_2_O]^−^, 191 [QA-H]^−^, **173** [QA-H-H_2_O]^−^, 163 [M-H-QA]^−^	[34]
9	Quercetin O-hexoside isomer 1	14.81	255, 355	463	445 [M-H-H_2_O]^−^, **301** [M-H-Hex]^−^, 179 [M-H-Hex-C_7_H_6_O_2_]^−^, 161 [M-H-Hex-C_7_H_8_O_3_]^−^	[34]
10	Quercetin O-rutinoside isomer 1	14.94	205, 280, 310	609	591 [M-H-H_2_O]^−^, 463 [M-H-Rha]^−^, **301** [M-H-Glu-Rha]^−^, 179 [M-H-Glu-Rha-C_7_H_6_O_2_]^−^	[35]
11	Naringenin O-hexoside	15.19	215, 280, 310	433	415 [M-H-H_2_O]^−^, 313 [M-H-C_4_H_8_O_4_]^−^, **271** [M-H-Hex]^−^, 151 [M-H-Hex-C_8_H_8_O]^−^	[35]
12	Rutin	15.21	210, 280, 320	609	591 [M-H-H_2_O]^−^, 463 [M-H-Rha]^−^, **301** [M-H-Glu-Rha]^−^, 179 [M-H-Glu-Rha-C_7_H_6_O_2_]^−^	[35]
13	Quercetin O-hexoside isomer 2	15.58	255, 355	463	445 [M-H-H_2_O]^−^, 343 [M-H-C_4_H_8_O_4_]^−^, **301** [M-H-Hex]^−^, 179 [M-H-Hex-C_7_H_6_O_2_]^−^	[35]
14	Apigenin O-hexoside	15.93	215, 280, 320	431	413 [M-H-H_2_O]^−^, 353 [?], 311 [M-H-C_4_H_8_O_4_]^−^, **269** [M-H-Hex]^−^	[35]
15	Kaempferol O-rutinoside	16.07	255, 350	593	575 [M-H-H_2_O]^−^, 327 [M-H-Pent-C_4_H_8_O_4_]^−^, **285** [M-H-Pent-Hex]^−^, 257 [M-H-Pent-Hex-CO]^−^	[36]
16	Quercetin O-hexoside isomer 3	16.12	255, 350	463	445 [M-H-H_2_O]^−^, 343 [M-H-C_4_H_8_O_4_]^−^, **301** [M-H-Hex]^−^, 179 [M-H-Hex-C_7_H_6_O_2_]^−^	[36]
17	Quercetin O-pentoside	16.74	265, 320	433	415 [M-H-H_2_O]^−^, 301 [M-H-Pent]^−^, 179 [M-H-Pent-C_7_H_6_O_2_]^−^, 151 [M-H-Pent-C_8_H_6_O_3_]^−^	[37]
18	Quercetin O-rhamnoside	16.89	255, 345	447	429 [M-H-H_2_O]^−^, 301 [M-H-Rha]^−^, 179 [M-H-Rha-C_7_H_6_O_2_]^−^, 151 [M-H-Rha-C_8_H_6_O_3_]^−^	[37]
19	Quercetin O-hexoside isomer 4	17.02	280, 320	463	445 [M-H-H_2_O]^−^, 343 [M-H-C_4_H_8_O_4_]^−^, **301** [M-H-Hex]^−^, 179 [M-H-Hex-C_7_H_6_O_2_]^−^	[37]
20	Phloridzin	17.57	220, 285	435	417 [M-H-H_2_O]^−^, 273 [M-H-Hex]^−^, 167 [M-H-C_13_H_16_O_6_]^−^	[38]
21	Kaempferol 3-*O*-rhamnoside	18.16	215, 265, 315	431	403 [M-H-CO]^−^, 327 [M-H-C_4_H_8_O_3_]^−^, **285** [M-H-Rha]^−^, 179 [M-H-Rha-C_7_H_6_O]^−^	[36]
22	Quercetin O-rutinoside isomer 2	19.01	220, 280, 320	609	591 [M-H-H_2_O]^−^, **463** [M-H-Rha]^−^, 343 [M-H-Rha-C_4_H_8_O_4_]^−^, 301 [M-H-Glu-Rha]^−^	[36]
23	Phloretin	22.28	220, 285	273	255 [M-H-H_2_O]^−^, **167** [M-H-C_7_H_6_O]^−^, 125 [M-H-C_9_H_8_O_2_]^−^	[36]

**Table 4:** Antiradical activity of AAL extract evaluated by DPPH, ABTS, and FRAP assays.

Antiradical Activity (µmol TE/g AAL DW ± SD)		
DPPH Assay	ABTS Assay	FRAP Assay
21.17 ± 2.30	38.82 ± 0.69	34.14 ± 3.83

The results are expressed as µmol TE per gram of AAL. Abbreviations: AALs, Annurca apple leaves; DPPH, 2,2diphenyl-1-picrylhydrazyl; ABTS, 2,20-azino-bis (3-ethylbenzothiazoline-6-sulfonic acid); FRAP, ferric reducing antioxidant power; TE, Trolox equivalent, DW, dry weight. Values are mean ± standard deviation (SD) of three repetitions.

## Data Availability

The data used to support the findings of this study are included in this article.

## Supplementary Materials

The following supporting information can be downloaded at: https://www.mdpi.com/article/10.3390/antiox11101933/s1, Figure S1: HPLC-FLD chromatogram of OAALE extract; Figure S2: Zoomed area of HPLC-FLD chromatogram of OAALE extract; Figure S3: HPLC-DAD chromatogram at 280 nm of OAALE extract; Figure S4: HPLC-DAD chromatogram at 360 nm of OAALE extract.
