# Supplementary material for: Optimization of Phlorizin Extraction from Annurca Apple Tree Leaves Using Response Surface Methodology
Source: Antioxidants (Basel). 2022 Sep 28;11(10):1933. doi: 10.3390/antiox11101933 (PMC9598179; doi:10.3390/antiox11101933)
Supplement: Supplementary file 1 [file antioxidants-11-01933-s001.zip › antioxidants-1863891-supplementary.pdf]

Supplementary material

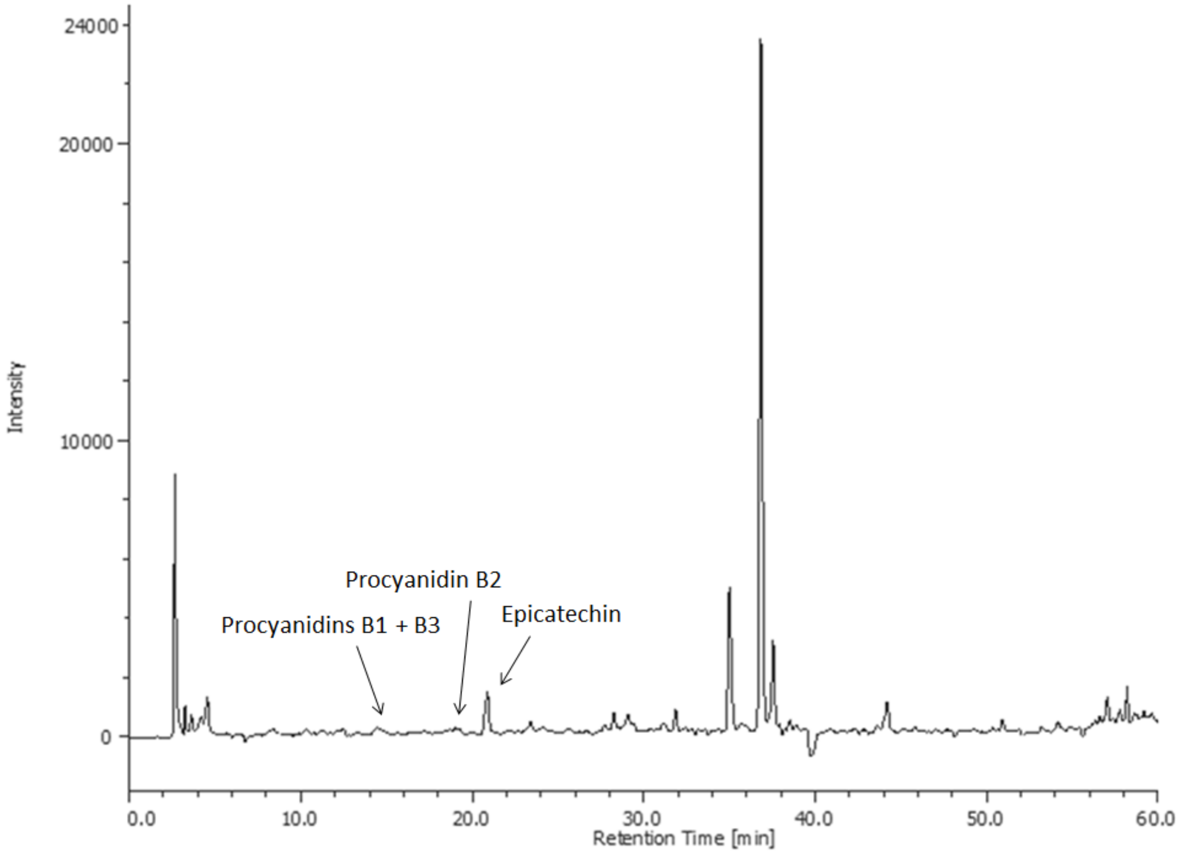

Figure S1. HPLC-FLD chromatogram of OAALE extract.

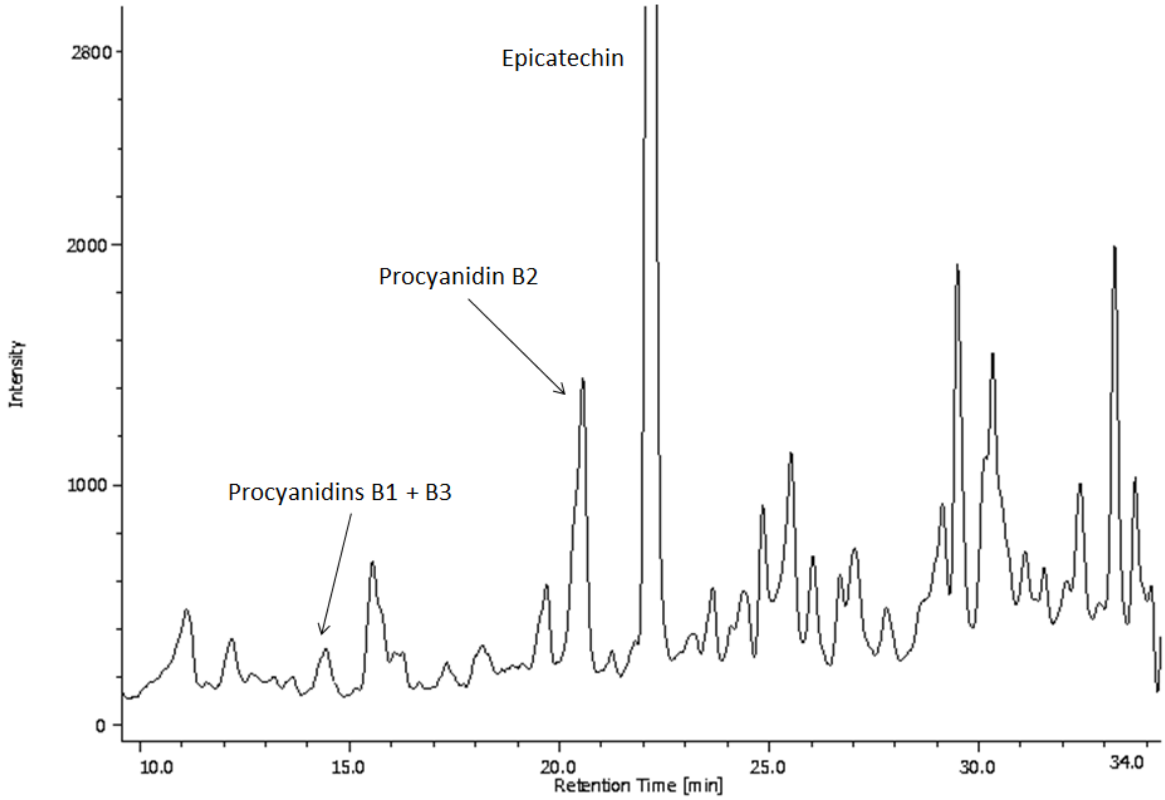

Figure S2. Zoomed area of HPLC-FLD chromatogram of OAALE extract.

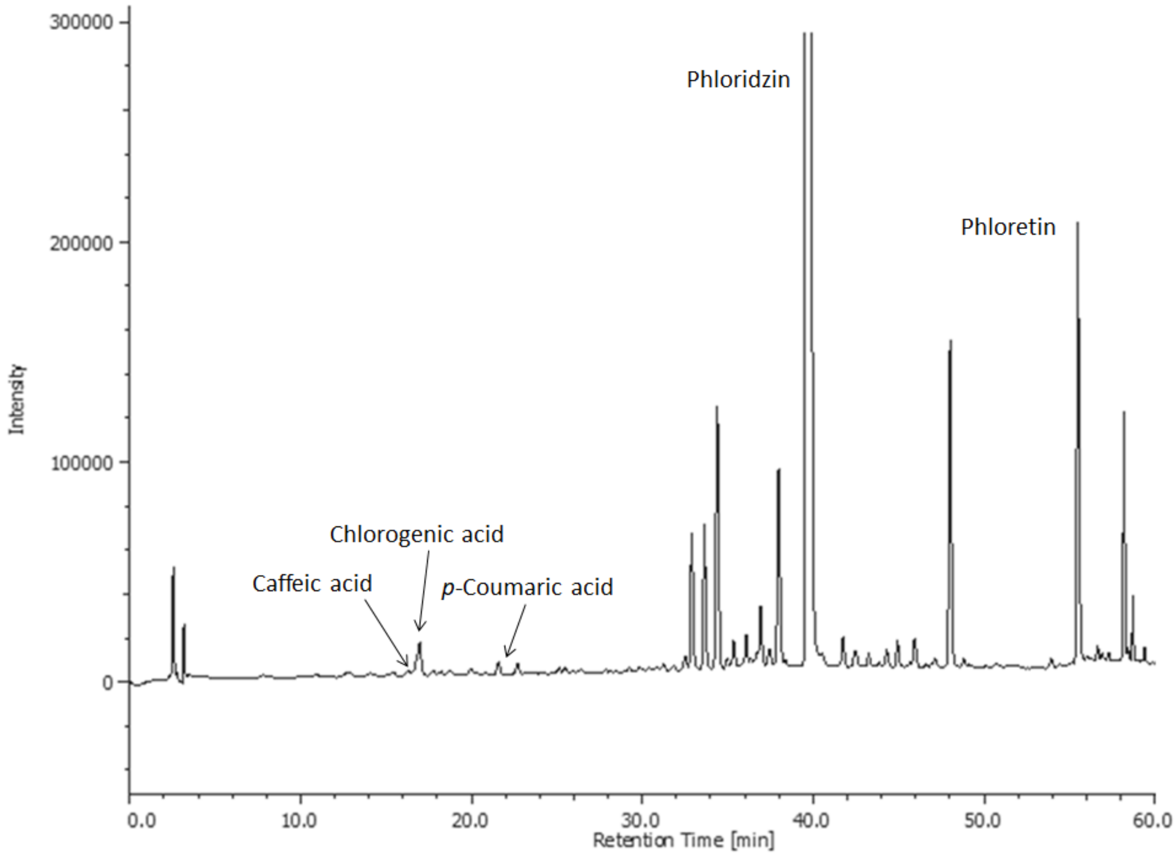

Figure S3. HPLC-DAD chromatogram at 280 nm of OAALE extract.

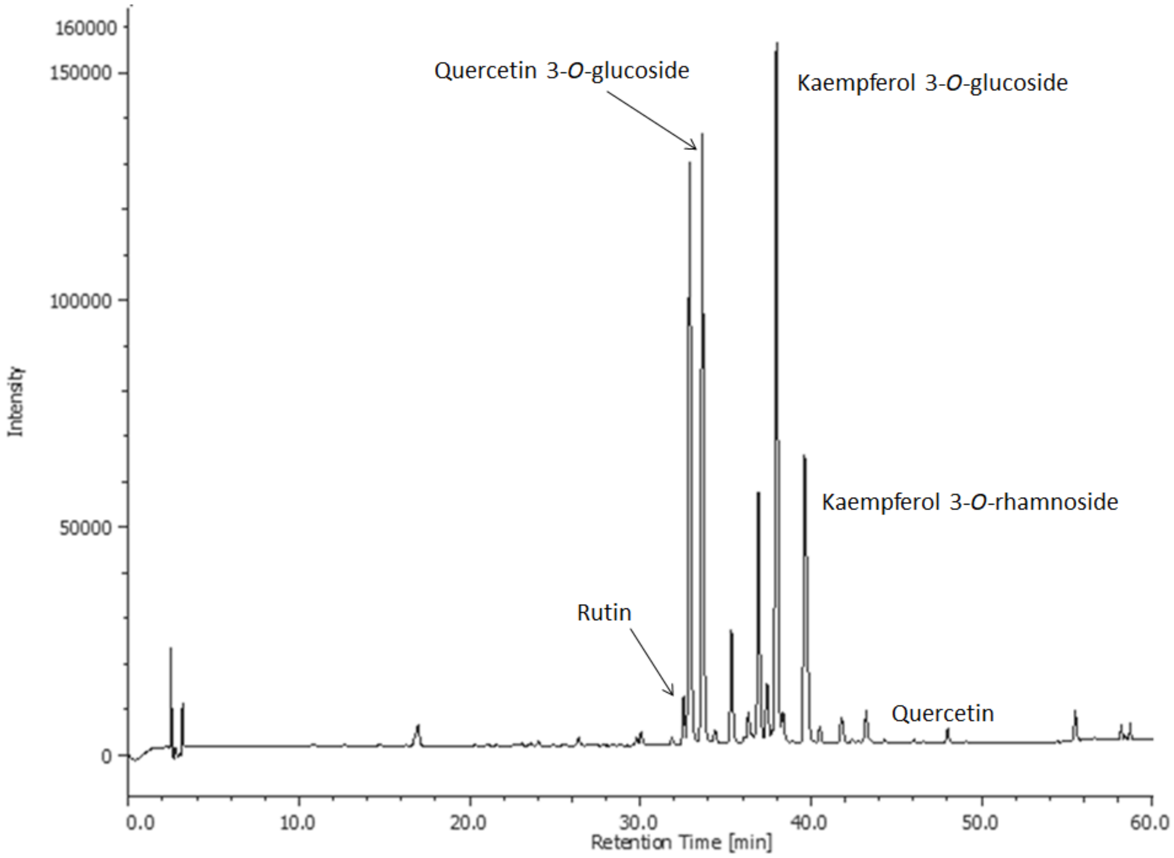

Figure S4. HPLC-DAD chromatogram at 360 nm of OAALE extract.
